# Age and Follicle-Stimulating Hormone as Variables Independently Associated with Serum Anti-Müllerian Hormone in Women Attending a Tertiary Gynaecology Clinic: A Retrospective Cross-Sectional Study Using Censoring-Aware Modelling

**DOI:** 10.3390/metabo16090610

**Published:** 2026-08-26

**Authors:** Mete Hakan Karalök, Bağnu Dündar, Ayhan Parmaksız, Tugba Elgün, Sevgi Koçyiğit Sevinç, Asiye Gök Yurttaş

**Affiliations:** 1 Department of Obstetrics and Gynecology, Faculty of Medicine, Istanbul Atlas University, Istanbul 34403, Türkiye; hakan.karalok@atlas.edu.tr; 2 Department of Biochemistry, Faculty of Medicine, Istanbul Atlas University, Istanbul 34403, Türkiye; bagnu.dundar@atlas.edu.tr; 3 Department of Biostatistics, Faculty of Medicine, Istanbul Health and Technology University, Istanbul 34445, Türkiye; ayhan.parmaksiz@istun.edu.tr; 4 Department of Medical Biology, Faculty of Medicine, Biruni University, Istanbul 34015, Türkiye; telgun@biruni.edu.tr; 5 Biruni University Research Center (B@MER), Biruni University, Istanbul 34015, Türkiye; 6 Department of Biophysics, Faculty of Medicine, Kutahya Health Sciences University, Kütahya 43100, Türkiye; sevgi.kocyigitsevinc@ksbu.edu.tr

**Keywords:** anti-Müllerian hormone, ovarian reserve, follicle-stimulating hormone, censored data, Tobit regression, Box–Cox transformation

## Abstract

**Objective**: Anti-Müllerian hormone (AMH) is the most widely used biochemical marker of ovarian reserve, but its associations with other reproductive hormones are usually examined one hormone at a time and without accounting for values reported at the assay floor. This study examined the associations between serum AMH and age, follicle-stimulating hormone (FSH), luteinising hormone (LH), estradiol, progesterone and prolactin in women attending a tertiary gynaecology clinic. **Materials and Methods**: In this retrospective, cross-sectional, single-centre study, the records of women who underwent serum AMH testing together with a reproductive hormone panel between 1 January 2020 and 31 December 2025 were reviewed. Women with polycystic ovary syndrome, primary ovarian insufficiency, pregnancy or previous ovarian surgery were excluded. Bivariate associations were assessed with Pearson and Spearman correlation coefficients on raw and Box–Cox-transformed variables. Because 12.82% (*n* = 15) of AMH results were left-censored at the analytical reporting floor of 0.02 ng/mL, a multivariable Tobit model with the censoring limit specified on the Box–Cox-transformed scale (λ = 0.309, threshold = −2.270) was fitted, with predictors selected a priori on clinical grounds. Sensitivity analyses examined the influence of high AMH values, an alternative transformation of AMH, and flexible modelling of age. **Results**: The analysis included 117 women aged 18–45 years. Mean AMH was 2.22 ± 2.24 ng/mL [median 1.61 ng/mL (Q1–Q3, 0.36–3.20)]. In bivariate correlation analysis, AMH showed statistically significant inverse associations with age (r = −0.376, *p* < 0.001) and FSH (r = −0.445, *p* < 0.001), whereas LH, estradiol, progesterone, and prolactin showed no statistically significant correlations with AMH (all *p* > 0.10). In the multivariable Tobit model, only age (β = −0.068; 95% CI [−0.117, −0.018]; *p* = 0.007) and transformed FSH (β = −3.582; 95% CI [−4.846, −2.317]; *p* < 0.001) remained independently associated with transformed AMH. LH, estradiol, progesterone, and prolactin were not independently associated with AMH in the multivariable model (all *p* > 0.05). The standardised association was greater for transformed FSH (β* = −0.559) than for age (β* = −0.234). The associations of age and FSH were consistent in direction and magnitude across all sensitivity analyses. **Conclusions**: In this selected clinical sample, age and FSH were the only variables independently associated with serum AMH after mutual adjustment. LH, estradiol, progesterone, and prolactin showed no statistically significant associations with AMH in either the unadjusted bivariate analyses or the multivariable model. The absence of independent associations for these hormones should be interpreted cautiously because hormone sampling was not standardised to menstrual cycle day, which may have introduced measurement variability. These findings describe associations within the study population and do not establish causal relationships, reference intervals, or a basis for modifying testing strategies. The analysis also highlights the importance of accounting for left-censoring when modelling AMH data containing a substantial proportion of results at the assay reporting floor.

## 1. Introduction

The assessment of female reproductive potential remains a central task in reproductive medicine. Anti-Müllerian hormone (AMH) has become the most widely used biochemical marker of ovarian reserve. AMH is a dimeric glycoprotein of the transforming growth factor-beta superfamily produced by the granulosa cells of preantral and small antral follicles [1,2], and its serum concentration reflects the size of the remaining growing follicle pool [3,4]. Unlike FSH and estradiol, AMH concentrations vary comparatively little across the menstrual cycle, which permits measurement on any cycle day [5,6].

AMH inhibits both the initial recruitment of primordial follicles and the cyclic selection of the dominant follicle and therefore acts as a negative regulator of follicular development [7]. Serum concentrations peak during the third decade of life and decline thereafter, reflecting progressive follicular attrition [8], becoming undetectable around menopause [9]. This trajectory underlies the use of AMH in predicting the timing of menopause, in stratifying ovarian response before assisted reproductive technology, and in monitoring gonadotoxic treatment [10,11,12,13].

Reproductive function is not governed by AMH in isolation. Within the hypothalamic–pituitary–ovarian axis, FSH, luteinising hormone (LH), estradiol, progesterone, prolactin and androgens jointly regulate follicular development, ovulation and corpus luteum function [14]. FSH drives antral follicle maturation, LH triggers ovulation and supports luteal function, and estradiol exerts negative feedback on FSH during the follicular phase before the midcycle surge that initiates ovulation [15]. Prolactin modulates gonadotrophin secretion and may suppress ovarian function when chronically elevated [16]. Androgens support early follicular growth at physiological concentrations, whereas androgen excess, as in polycystic ovary syndrome (PCOS), is associated with follicular arrest [17].

An inverse association between serum AMH and basal FSH is well described: as follicular output falls, reduced inhibin B- and estradiol-mediated negative feedback is accompanied by a rise in pituitary FSH secretion [18]. Conversely, the elevated AMH concentrations characteristic of PCOS frequently accompany an increased LH/FSH ratio [19]. The relationship between AMH and estradiol is less consistent across studies, partly because of cycle-dependent variation in estradiol [20]. Positive associations between AMH and testosterone have been reported in PCOS [21], and reproductive and lifestyle factors, including body mass index and smoking are also associated with AMH concentrations [22].

Two methodological issues recur in this literature. First, most studies analyse untransformed, markedly skewed hormone distributions with linear correlation methods, which can misrepresent the strength of an association. Second, AMH datasets drawn from clinical populations regularly contain a substantial proportion of results at the lower reporting limit of the assay. Substituting a fixed value for such results or analysing them as though they were measured, produces biassed estimates; methods for left-censored data are required instead [23,24]. AMH measurement is further complicated by differences between assay generations and platforms, which limit the comparability of absolute concentrations across studies [25,26].

Against this background, the present study examined the associations between serum AMH and age, FSH, LH, estradiol, progesterone and prolactin in a retrospective cohort of women attending a tertiary gynaecology outpatient clinic, using transformation to address skewness and a censored-outcome model to accommodate results at the assay floor. The aim was to determine which of these routinely measured variables remain associated with AMH after mutual adjustment within this dataset and not to derive reference intervals or normative data.

## 2. Materials and Methods

### 2.1. Study Design and Setting

This was a retrospective, cross-sectional, single-centre study conducted at a tertiary university hospital. The study population comprised women who underwent serum AMH testing as part of routine clinical care between 1 January 2020 and 31 December 2025. Records were identified through the hospital information management system and the laboratory information system. Reporting follows the STROBE statement for observational studies [27].

To avoid ambiguity between the clinical period from which the data originate and the period during which the study itself was conducted, the two are stated separately. All laboratory measurements were performed during routine care within the clinical window given above. Ethics committee approval was granted on 22 June 2026 (Ethics Committee Decision No. 06/09). The relevant records were subsequently extracted from the hospital information systems, and the statistical analyses were performed thereafter. No data were collected prospectively, and no patient contact took place at any stage.

### 2.2. AMH Assay and Analytical Characteristics

Serum anti-Müllerian hormone (AMH) concentrations were measured using the VIDAS^®^ AMH assay (Ref. 417011; bioMérieux, Marcy-l’Étoile, France) on the VIDAS^®^ 3 automated immunoassay platform, based on the enzyme-linked fluorescent assay (ELFA) principle. The assay is validated for the quantitative determination of AMH in human serum and lithium-heparin plasma. In the present study, serum samples were used for AMH measurement, with a required sample volume of 200 μL.

According to the manufacturer’s specifications, the validated analytical measurement range of the VIDAS^®^ AMH assay is 0.02–9.00 ng/mL. The limit of detection (LoD) is 0.01 ng/mL, whereas the lower limit of quantification (LoQ) is 0.02 ng/mL. Accordingly, 0.01 ng/mL represents the analytical detection limit, whereas 0.02 ng/mL represents the lowest concentration within the validated quantitative measurement range. Samples with AMH concentrations exceeding 9.00 ng/mL can be diluted according to the manufacturer’s recommended procedure and reanalysed, with the final concentration calculated using the appropriate dilution factor.

The manufacturer’s analytical precision evaluation included five AMH concentration levels (0.22, 1.08, 2.99, 5.45, and 7.37 ng/mL). Reported coefficients of variation (CVs) for repeatability (within-run precision) ranged from 4.1% to 4.8%, intra-lot CVs ranged from 6.6% to 8.2%, and intra-laboratory precision CVs ranged from 8.3% to 10.6%. The coefficient of variation between reagent lots did not exceed 11%.

Calibration and internal quality control procedures were performed in accordance with the manufacturer’s instructions. The VIDAS^®^ AMH assay uses the S1 calibrator and C1 control, with calibration and control validity specified by the manufacturer. Calibration was performed according to the manufacturer’s recommendations and repeated when required, including following reagent-lot changes. Patient results were accepted only when internal quality control results fulfilled the laboratory’s predefined acceptance criteria. Other reproductive and pituitary hormones were measured using validated automated immunoassays according to the respective manufacturers’ instructions, with routine calibration and internal quality control procedures performed in accordance with the laboratory’s established quality-management procedures.

The same VIDAS^®^ AMH assay and VIDAS^®^ 3 platform were used throughout the study period, with no change in the AMH assay platform or reagent generation during the study period. Because the validated lower limit of quantification of the AMH assay was 0.02 ng/mL, 0.02 ng/mL was used as the censoring threshold in the revised Tobit analysis. The previous use of 0.01 ng/mL, corresponding to the assay LoD rather than the LoQ, was corrected in the revised statistical analysis.

Serum AMH concentrations exceeding 10 ng/mL were considered high upper-tail observations for analytical purposes and were not interpreted as evidence of polycystic ovary syndrome (PCOS) or other pathology. Although elevated AMH concentrations may occur in women with PCOS, AMH alone is not sufficient to establish a diagnosis of PCOS, and high AMH values may also be observed in women without PCOS. Therefore, the threshold of 10 ng/mL was used solely as a laboratory-based eligibility criterion to limit the potential influence of extreme upper-tail observations on the primary statistical analysis and was not applied as a clinical diagnostic threshold.

Records with AMH > 10 ng/mL were excluded from the primary analysis to reduce the potential influence of extreme upper-tail observations on regression estimates. The clinical and endocrine characteristics of these women are presented in Supplementary Table S2. To assess the robustness of the findings to the inclusion of these observations, all primary analyses were repeated with participants with AMH > 10 ng/mL retained in the dataset as a sensitivity analysis. The results were compared with those of the primary analysis.

### 2.3. Eligibility Criteria and Dataset Audit

Eligible records were those of women who had a serum AMH measurement together with a reproductive hormone panel obtained concurrently from the same venipuncture blood sample during routine clinical evaluation. Eligible records were identified through two sequential screening stages, with the initial electronic/clinical screening involving a review of available ICD-10 diagnostic codes and clinical records to identify and exclude women with documented conditions that could affect ovarian reserve or reproductive hormone concentrations. These clinical exclusions included systemic endocrine disorders, pregnancy, previous bilateral oophorectomy or ovarian surgery, documented polycystic ovary syndrome (PCOS), primary ovarian insufficiency (POI), and other established syndromic or ovarian conditions expected to influence AMH or reproductive hormone concentrations. The ICD-10-coded clinical diagnoses recorded by the treating physicians were used solely for eligibility screening and exclusion purposes and were therefore not included as analytical variables or tabulated in the Results.

Records remaining after the initial clinical screening underwent a second, laboratory-based eligibility screening. Eligible records were required to include a serum AMH measurement and a complete reproductive hormone panel obtained concurrently from the same venipuncture blood sample during routine clinical evaluation. Additional laboratory and demographic eligibility criteria were age 18–45 years, serum FSH ≤ 30 mIU/mL, and serum AMH ≤ 10 ng/mL. Records with incomplete or internally inconsistent laboratory data were excluded. Thus, the 1873 records represented the initial screened population, whereas the final analytical cohort consisted of 117 women who fulfilled all clinical, demographic, and laboratory eligibility criteria after completion of the sequential screening process.

Following the initial review of the manuscript, the complete dataset was re-audited against the source records to verify eligibility and cohort construction. Screening errors and out-of-range records, including records from women younger than 18 or older than 45 years, were systematically identified and removed. The age eligibility criterion was therefore strictly restricted to women aged 18–45 years in the final analytical cohort (*n* = 117).

For eligibility screening, an FSH concentration > 30 mIU/mL was used as an operational biochemical exclusion criterion to identify women with evidence of overt hypergonadotropic ovarian dysfunction. This threshold was used as a pragmatic laboratory-based screening floor and was not considered equivalent to a formal diagnosis of primary ovarian insufficiency (POI). Documented clinical diagnoses of POI were identified separately during the initial clinical/ICD-10 screening stage. Because this was a retrospective analysis of routinely collected clinical laboratory data, serial FSH measurements, longitudinal menstrual or amenorrhea records, and prospective clinical assessments required to establish a formal diagnosis of POI were not consistently available. Therefore, FSH > 30 mIU/mL was applied solely as a laboratory-based exclusion criterion during the eligibility screening process rather than as a diagnostic criterion for POI.

The AMH threshold of ≤10 ng/mL was applied as a laboratory-based eligibility criterion for cohort construction and was not interpreted as a diagnostic criterion for PCOS. In particular, an AMH concentration > 10 ng/mL was not considered evidence of PCOS in the absence of a documented clinical diagnosis. The threshold was used solely to define the analytical cohort and to limit the influence of extreme upper-tail AMH observations on the primary analysis. The potential influence of these observations was additionally evaluated in a sensitivity analysis in which participants with AMH > 10 ng/mL were retained.

### 2.4. Data Collection and Laboratory Parameters

Data were retrospectively obtained from the Hospital Information Management System (HIMS) and Laboratory Information System (LIS). Clinical diagnoses were identified using ICD-10 diagnostic codes recorded by the treating physicians. For each eligible participant, an anonymized study code was generated, and the following variables were extracted using a standardised data collection form specifically developed for this study: age, serum AMH level, FSH, LH, estradiol, progesterone, prolactin, additional available reproductive hormone parameters, clinical diagnosis, and date of laboratory measurement.

Menstrual cycle characteristics, including cycle regularity and the cycle day or phase at the time of blood sampling, were not systematically recorded in the source records and therefore could not be reliably extracted for most participants. Consequently, hormone sampling occurred at unstandardised cycle days during routine clinical care. The potential implications of this lack of menstrual cycle standardisation for the cycle-dependent hormones (estradiol, progesterone, and LH) are addressed in the Limitations.

All laboratory measurements had been performed as part of routine clinical care in the hospital biochemistry laboratory using validated automated immunochemical assay methods according to the manufacturers’ recommendations and internal quality control procedures. No additional blood sampling, patient intervention, questionnaire administration, or direct patient contact was performed during the study. Personal identifiers, including names and national identification numbers, were excluded from the dataset to ensure patient confidentiality.

### 2.5. Statistical Analysis

Continuous variables are summarised as mean ± standard deviation, median with interquartile range, and minimum–maximum values (*n* = 117), with the number of available observations reported for each variable.

Serum hormone concentrations were markedly right-skewed and were therefore analysed both untransformed and after Box–Cox power transformation [28]. The transformation was defined as y(λ) = (y^λ^ − 1)/λ for λ ≠ 0 and y(λ) = ln(y) for λ = 0. For each variable, the optimal power parameter (λ) was estimated by maximum likelihood: the profile log-likelihood was first evaluated over a coarse grid from −2.000 to +2.000 to locate the neighbourhood of the optimum, and the optimum was then identified over a fine grid in increments of 0.001, using the boxcox function of the MASS package; the value maximising the log-likelihood was retained. Estimated λ parameters were: AMH (λ = 0.309), FSH (λ = −0.358), LH (λ = 0.207), estradiol (λ = −0.484), progesterone (λ = −0.206), and prolactin (λ = 0.064); age was not transformed. λ estimates and their 95% confidence intervals are given in Supplementary Table S3.

Bivariate associations were quantified with Pearson correlation coefficients on both scales and with Spearman rank correlation coefficients.

Because 12.82% (*n* = 15) of AMH results lay at the analytical reporting floor (0.02 ng/mL), the multivariable analysis used a Tobit model for a left-censored outcome [23,24], fitted by maximum likelihood using the AER package. The dependent variable was Box–Cox-transformed AMH. Critically, the censoring limit supplied to the model was the Box–Cox transform of 0.02 ng/mL evaluated at λ = 0.309 (BC_AMH_ = −2.270), rather than the untransformed value, so that the censoring point corresponded to the scale on which the outcome was modelled.

Predictors were specified a priori on clinical and physiological grounds rather than selected according to their bivariate association with the outcome, because data-driven selection based on a correlation threshold produces unstable estimates and standard errors that are conditional on the selection step [29]. The prespecified model therefore included age, FSH_bc, LH_bc, estradiol_bc, progesterone_bc, and prolactin_bc, each on its transformed scale where applicable; prolactin, which had previously been omitted on the basis of its bivariate coefficient, was retained.

Because the decline of AMH with age is known to be non-linear, age was additionally modelled with a restricted cubic spline with knots at the 10th, 50th, and 90th percentiles, and the linear and spline specifications were compared by likelihood ratio test; the linear specification was retained only if it was not rejected.

Model diagnostics indicated no evidence of problematic multicollinearity, with variance inflation factors (VIF) ranging from 1.044 to 1.349 (Supplementary Table S1). Diagnostics further comprised inspection of standardised residuals against fitted values, quantile–quantile plots of residuals (Supplementary Figure S1), and assessment of influential observations. Effect estimates are reported as unstandardised coefficients (β) with 95% confidence intervals. Because predictors were transformed with different λ values, unstandardized coefficients are not directly comparable across predictors; average marginal effects on the AMH_bc scale and standardized beta coefficients (β*), obtained after scaling each predictor to unit standard deviation, are reported in Supplementary Table S4 and used for direct effect comparisons across transformed predictors.

Sensitivity analyses were: (a) inclusion of the seven women with AMH above 10 ng/mL; (b) log-transformation of AMH in place of the Box–Cox transformation; and (c) flexible modelling of age with restricted cubic splines;

All tests were two-sided with a significance threshold of 0.05. Analyses were performed in R version 4.5.1 (R Foundation for Statistical Computing, Vienna, Austria) using the packages AER (v1.2.15), MASS (v7.3.65), Hmisc (v5.2.4), rms, and ggplot2 (v4.0.3).

## 3. Results

### 3.1. Participants and Baseline Characteristics

**Figure 1:**
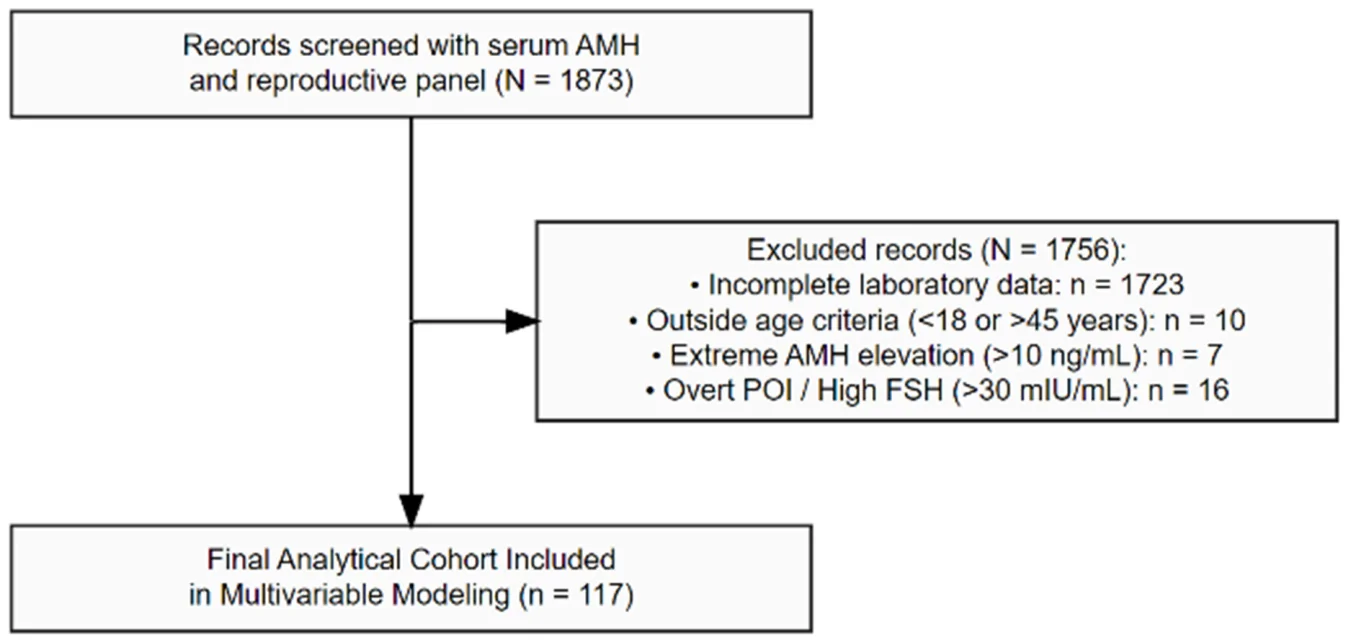
Flow diagram illustrating the sequential clinical and laboratory-based screening process, exclusion criteria, and final selection of the analytical cohort (*n* = 117).

Following the dataset audit and eligibility screening, the study cohort was established through sequential clinical and laboratory-based screening. An initial pool of 1873 records was screened using available clinical records and diagnostic (ICD-10) coding to identify and exclude records with documented systemic endocrine disorders, pregnancy, previous bilateral oophorectomy/ovarian surgery, and established syndromic conditions. The remaining records were subsequently subjected to laboratory-based eligibility screening, including completeness of the reproductive hormone panel, restriction to 18–45 years, FSH ≤ 30 mIU/mL, and AMH ≤ 10 ng/mL. After completion of this sequential screening process, the final harmonised analytical cohort comprised 117 women (Figure 1).

Exactly 12.82% (*n* = 15) of patients presented with serum AMH levels at the analytical reporting floor (0.02 ng/mL, corresponding to BC_AMH_ = −2.270). The baseline clinical and demographic characteristics of the cohort are detailed in Table 1. Mean age was 32.98 ± 5.56 years. Hormone concentrations were right-skewed, most notably for estradiol (mean 109.21 ± 226.20 pg/mL; median 48.00 pg/mL) and progesterone (mean 4.05 ± 6.47 ng/mL; median 0.30 ng/mL), with mean values consistently exceeding medians across all analytes.

Table 1 presents all variables available for analysis; apart from age and the hormonal parameters, no further clinical or anthropometric data (including body mass index, smoking status, infertility status or contraceptive use) were recorded in the source dataset (see Section 6).

### 3.2. Bivariate Associations

In the untransformed data matrix, age (r = −0.376, *p* < 0.001; ρ = −0.317, *p* = 0.0005) and serum FSH (r = −0.445, *p* < 0.001; ρ= −0.503, *p* < 0.001) demonstrated significant inverse associations with serum AMH. LH (*p* = 0.134), estradiol (*p* = 0.218), progesterone (*p* = 0.533), and prolactin (*p* = 0.572) failed to show formal linear or rank-based correlations.

**Figure 2:**
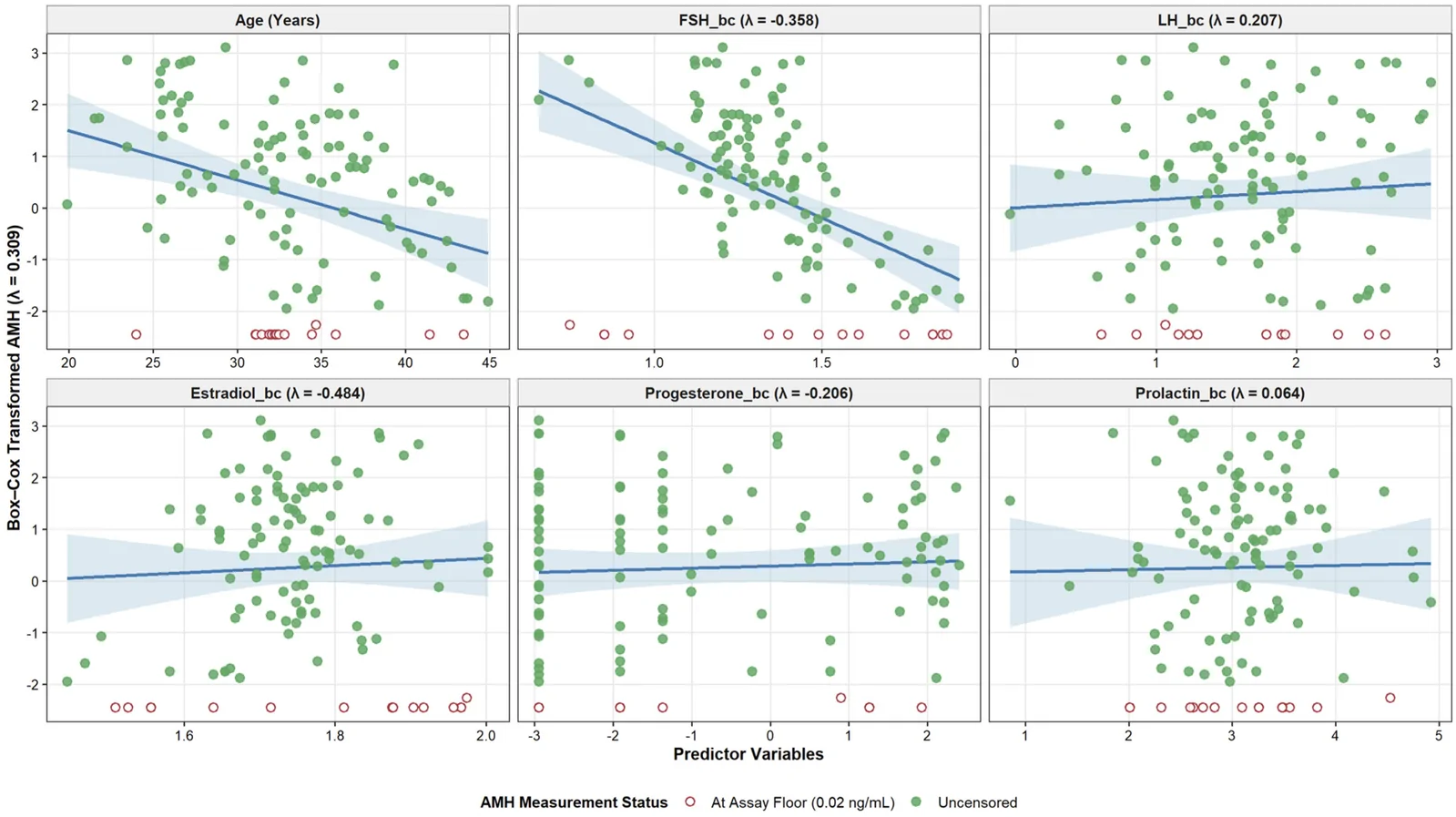
Bivariate scatterplots illustrating the relationships between Box–-Cox-transformed serum AMH and independent clinical/endocrine predictors (age, FSH_bc, LH_bc, Estradiol_bc, Progesterone_bc, and Prolactin_bc).

Following Box–Cox transformations (λ_AMH_ = 0.309, λ_FSH_ = −0.358, λ_LH_ = 0.207, λ_Estradiol_ = −0.484, λ_Progesterone_ = −0.206, λ_Prolactin_ = 0.064), AMH_bc maintained significant linear correlations with age (r = −0.330, *p* < 0.001) and FSH_bc (r = −0.454, *p* < 0.001). The crude associations of all other transformed hormones remained statistically non-significant (*p* > 0.50). These relationships are illustrated in Figure 2, where the near-flat regression slopes and wide confidence bands for LH_bc, estradiol_bc, progesterone_bc, and prolactin_bc visually reflect the absence of a significant linear association, consistent with their retention in the multivariable model on a priori clinical grounds rather than on the basis of bivariate significance. Correlation parameters for both untransformed and transformed variables are presented in Table 2 and Table 3, respectively.

### 3.3. Multivariable Tobit Regression Model

The multivariable Tobit model evaluated all hormonal variables simultaneously while explicitly accounting for left-censoring at BC_AMH_ = −2.270. VIF values indicated no evidence of problematic multicollinearity (VIF range: 1.044–1.349).

Model diagnostics for the multivariable Tobit regression were systematically evaluated using standardised residuals (Supplementary Figure S1). The residuals-versus-fitted plot (Panel A) confirmed overall model linearity, with residuals randomly dispersed around the zero-horizontal line. The normal Q–Q plot (Panel B) demonstrated close adherence to normality across uncensored observations; the deviation observed at the lower tail reflects the expected pattern arising from the 15 left-censored observations at the assay floor (0.02 ng/mL). The scale–location plot (Panel C) indicated stable residual variance across fitted outcome levels, consistent with homoscedasticity. The residuals-versus-index plot (Panel D) showed no evidence of influential outliers, with standardised residuals falling within ±3 standard deviations. These diagnostics support the distributional assumptions underlying the specified Tobit model.

In the mutually adjusted model, age (β = −0.068, *p* = 0.007) and transformed FSH (β = −3.582, *p* < 0.001) emerged as the sole statistically significant independent variables inversely associated with AMH (Table 4). Standardised beta coefficients indicated a larger standardised association of FSH with AMH (β* = −0.559) compared with age (β* = −0.234), with the magnitude of the association being approximately 2.4-fold greater for FSH. LH, estradiol, progesterone, and prolactin provided no additional independent explanatory power (*p* > 0.05).

### 3.4. Sensitivity and Model Specification Analyses

To evaluate the robustness of the primary multivariable Tobit model and assess sensitivity to structural assumptions, four distinct model specifications were compared (Table 5). Across all specifications, the primary independent inverse associations of age and FSH with serum AMH remained consistent in direction and magnitude, confirming the stability of the core biological findings.

Incorporating participants with extreme AMH concentrations (>10 ng/mL, *n* = 124) shifted the regression slopes for age (β = −0.087, 95% CI: [−0.138, −0.037]) and FSH (β = −3.874, 95% CI: [−5.196, −2.552]) and caused LH to reach statistical significance (β = 1.142, *p* = 0.005). Rather than reflecting physiological regulation across the general reproductive spectrum, this artifactual significance demonstrates substantial regression leverage exerted by a small number of extreme upper-tail observations. Restricting the primary cohort to AMH ≤ 10 ng/mL (*n* = 117) therefore mitigates high-leverage distortion of the multivariable covariance structure while evaluating non-syndromic ovarian ageing dynamics.

Applying a standard natural logarithmic transformation (λ = 0, *n* = 117) over-compressed the upper tail of the AMH distribution and widened the confidence interval for age (β = −0.064, 95% CI: [−0.127, −0.001], *p* = 0.048), with the upper bound approaching the null value. The direction and approximate magnitude of the association were unchanged, but the estimate was appreciably less precise than under the empirically optimised Box–Cox parameter (λ = 0.309, *p* = 0.007), which better stabilised residual variance. The core finding for age was therefore robust to the choice of transformation, although a fixed logarithmic transformation yielded a less efficient estimate.

Finally, age was modelled flexibly using a 3-knot restricted cubic spline (RCS). The coefficient for the linear spline basis (β_Age_ = −0.125) was estimated on a different basis from the linear model and is not directly comparable with it; the estimate for FSH_bc was essentially unchanged (β = −3.571 versus −3.582 in the primary model), indicating that flexible modelling of age did not alter the FSH–AMH association. A likelihood ratio test showed no statistically significant improvement in fit over the linear specification (χ^2^ = 2.61, *p* = 0.2663). In accordance with the principle of parsimony, a linear functional form for age was retained in the primary model.

## 4. Discussion

In this retrospective cross-sectional study of women attending a tertiary gynaecology clinic, age and FSH were the only variables that remained independently associated with serum AMH after mutual adjustment within a model that explicitly accommodated results at the analytical reporting floor. LH, estradiol, progesterone and prolactin showed no statistically significant bivariate association with AMH, whether analysed on the raw or Box–Cox-transformed scale, and none reached conventional significance after adjustment for age and FSH, although the estimate for estradiol_bc lay close to the conventional threshold (*p* = 0.055).

The inverse association between age and AMH, and its persistence after adjustment for FSH, is consistent with the established trajectory of follicular attrition with age [8,9]. That age remained independently associated with AMH after adjustment for FSH suggests that the age-related decline in AMH is not fully captured by the gonadotrophin feedback loop and reinforces the case for age-specific interpretation of AMH values rather than reliance on a single universal threshold. A recent large cohort study spanning nearly 23,000 women constructed an age-stratified AMH nomogram and reported that the sharp decline in AMH levels, particularly after age 36, underscores the need for timely fertility evaluation, especially in populations at higher risk of diminished ovarian reserve, a finding that closely parallels the age dependence observed in the present cohort [10,13,30,31].

FSH showed the most consistent association with AMH across analytical frameworks. On the raw scale, the Pearson coefficient (r = −0.445) was smaller in magnitude than the Spearman coefficient (ρ = −0.503), a pattern consistent with a monotonic but non-linear relationship. Box–Cox transformation moved the Pearson coefficient towards the rank-based estimate (r = −0.454), although a residual difference remained; the corresponding convergence was more complete for age, where the Pearson coefficient shifted from −0.376 to −0.330 against a Spearman coefficient of −0.317. This pattern indicates that linear correlation applied to untransformed, skewed hormone data does not fully capture the FSH–AMH relationship and that rank-based or transformation-based estimates should be reported alongside it. The direction of the association is consistent with the classical model in which declining follicular output reduces inhibin B- and estradiol-mediated negative feedback, accompanied by a compensatory rise in FSH [18]. It is nonetheless worth noting that basal FSH is widely regarded as a comparatively late and insensitive marker of declining ovarian reserve; as summarised in a recent review, FSH’s limited predictive value is illustrated by NHANES III data showing that most women aged 40–44 years still had FSH concentrations within the conventionally normal range, despite ovarian factors typically being rate-limiting at this age, and AMH is generally considered to outperform FSH as a predictor of ovarian response [32]. This body of evidence is compatible with, rather than contradictory to, the present findings: the association we observed describes how AMH and FSH co-vary within this cohort, not the relative clinical sensitivity of either marker in detecting diminished reserve. Because the predictors in this analysis were transformed on different scales, the relative magnitude of the FSH and age coefficients cannot be inferred from the unstandardised estimates; standardised comparisons are reported separately in Supplementary Table S4, where the standardised association was greater in magnitude for FSH_bc (β* = −0.559) than for age (β* = −0.234).

The absence of an independent association for LH, estradiol and progesterone after adjustment admits two explanations that this dataset cannot distinguish. The first is that their crude associations with AMH, where present in other populations, largely reflect variance shared with the age–FSH axis rather than independent biological information [14,18]. The second, and equally plausible, explanation is measurement variability: FSH, LH, estradiol and progesterone vary substantially across the menstrual cycle, and samples in this study were obtained during routine clinical care rather than on a standardised cycle day. Non-differential measurement error of this kind attenuates observed associations towards the null, so the absence of significant associations for the cycle-dependent hormones may partly reflect the timing of sampling rather than a true absence of a biological relationship. This interpretation is consistent with the previously reported inconsistency of the AMH–estradiol relationship across studies [20,25]. No inference about the clinical value of measuring these hormones can be drawn from this analysis. This caution is consistent with current guidance, which emphasises that ovarian reserve markers quantify the size of the remaining follicle pool rather than reproductive potential and that a diminished AMH concentration is in itself a poor predictor of natural fertility [12,33].

Prolactin was not associated with AMH in bivariate analysis and was not independently associated with AMH in the adjusted model. Within the physiological range represented in this cohort, prolactin does not appear to relate meaningfully to AMH concentrations; associations reported previously may be confined to pathological hyperprolactinaemic states [16], which were largely absent here.

Two methodological points merit emphasis. First, the marked skewness of the raw hormone distributions indicates that correlation analyses based on untransformed endocrine data may not adequately represent the underlying associations. This was most evident for age, where the Pearson coefficient converged closely on the rank-based estimate after transformation, and was present to a lesser degree for FSH [28,29]. Second, left-censoring at the assay floor is a structural feature of clinical AMH datasets that include women with severely diminished ovarian reserve and is frequently disregarded in the literature. Ordinary least-squares regression applied to such data yields biassed coefficients; a model for censored outcomes is the appropriate alternative [23,24]. It should be noted, however, that the estimated scale parameter of a Tobit model quantifies residual dispersion and does not in itself constitute evidence of model fit, precision, or adequate accommodation of heteroscedasticity; this was instead assessed through the residual diagnostics summarised in Supplementary Figure S1.

This study has several important limitations. The cohort consists of women attending a tertiary gynaecology clinic who underwent both AMH testing and an extended hormone panel and therefore constitutes a highly selected clinical sample. It is not representative of the general population of women of reproductive age, and the findings cannot be generalised beyond comparable clinical settings. Consequently, the study does not provide normative or population-specific data and does not establish reference intervals, which would require a defined reference population, prespecified health criteria, age stratification and an appropriate reference-interval methodology [34]. Second, the design is retrospective and cross-sectional, so the observed associations are adjusted associations within a single dataset and cannot establish determinants, biological independence or causal relationships. Third, hormone measurements were not standardised to menstrual cycle day, which limits the interpretation of the associations of the cycle-dependent hormones as discussed above. Fourth, information on indication for testing, infertility status, contraceptive and fertility treatment use, body mass index and smoking was unavailable, raising the possibility of residual confounding [16]. Fifth, women with PCOS were excluded, which improves internal validity by removing a condition in which the AMH–LH/FSH relationship differs substantially but limits generalisability to this subgroup, which is common in the source population [35]. Sixth, total testosterone was not available for the analysed cohort, and the hormone panel examined here is therefore not a complete reproductive hormone profile. Seventh, the study was conducted in a single centre with a single laboratory, and AMH results are known to differ between assay generations and platforms, which limits the transferability of the absolute concentrations reported here [25,26]. Finally, the study did not evaluate diagnostic performance, treatment outcomes or cost-effectiveness, and no recommendation regarding the composition of hormonal testing panels can be derived from these data.

## 5. Conclusions

In this retrospective cohort of women attending a tertiary gynaecology clinic, age and FSH were the only variables independently associated with serum AMH after mutual adjustment in a model that explicitly accounted for left-censoring at the analytical reporting floor. In bivariate analyses, AMH showed significant inverse associations with age and FSH, whereas LH, estradiol, progesterone, and prolactin showed no statistically significant associations with AMH. Consistent with these findings, only age and FSH remained independently associated with AMH in the multivariable model, while LH, estradiol, progesterone, and prolactin were not independently associated with AMH.

Because hormone sampling was not standardised to menstrual cycle day, potential measurement variability related to cycle timing cannot be excluded, particularly for the cycle-dependent hormones estradiol, progesterone, and LH. Therefore, the absence of statistically significant associations for these hormones should be interpreted cautiously and should not be taken as evidence of the absence of biological relationships.

These findings describe associations within the selected clinical population studied here and should not be interpreted as reference intervals, diagnostic thresholds, causal relationships, or a basis for revising routine hormonal testing panels. More broadly, this analysis illustrates two methodological considerations of relevance to endocrine datasets: the value of variance-stabilising transformation when analysing skewed hormone concentrations and the importance of censoring-aware modelling when a substantial proportion of AMH results lie at the assay reporting floor.

## 6. Limitations

A further limitation is that the retrospective nature of the dataset limited the clinical characterisation of ovarian function. Although an FSH concentration > 30 mIU/mL was used as a pragmatic biochemical exclusion criterion to screen for overt hypergonadotropic states, this threshold should not be interpreted as a formal diagnosis of primary ovarian insufficiency (POI). Serial FSH measurements, longitudinal menstrual or amenorrhea records, and comprehensive prospective clinical assessments were not consistently available in the retrospective dataset. Accordingly, women with a documented clinical diagnosis of POI were excluded during the clinical screening stage, whereas an FSH concentration > 30 mIU/mL served separately as a laboratory-based exclusion criterion. This distinction should be considered when interpreting the study population and its generalizability.

In addition, although all analysed reproductive and pituitary hormones were measured concurrently from the same single venipuncture blood draw, the retrospective dataset did not consistently contain detailed information regarding menstrual cycle phase at the time of sampling. Therefore, potential physiological variation related to cycle timing, particularly for gonadal hormones, cannot be completely excluded.

Menstrual cycle timing represents an additional limitation of this retrospective study. Menstrual cycle regularity and the exact cycle day or phase at the time of blood sampling were not systematically recorded in the source clinical records and therefore could not be reliably determined for most participants. Consequently, hormone measurements were obtained at non-standardised points of the menstrual cycle as part of routine clinical care. This may have introduced additional biological variability, particularly for cycle-dependent hormones such as estradiol, progesterone, and LH, and may have attenuated or obscured their associations with AMH. Therefore, the findings for these hormones should be interpreted cautiously, and prospective studies with standardised early follicular-phase sampling are warranted to minimise the potential influence of menstrual cycle timing.

## Figures and Tables

**Table 1:** Baseline Clinical and Demographic Characteristics (*n* = 117).

Variable	Mean ± SD	Median (Q1–Q3)	Min–Max
AMH (ng/mL)	2.22 ± 2.24	1.61 (0.36–3.20)	0.01–8.80
Age (Years)	32.98 ± 5.56	32.83 (29.23–36.70)	19.93–44.91
Estradiol (pg/mL)	109.21 ± 226.20	48.00 (34.00–71.00)	12.00–1355.00
FSH (mIU/mL)	7.53 ± 4.61	6.30 (4.84–8.11)	2.11–25.12
LH (mIU/mL)	4.45 ± 2.03	4.25 (2.83–5.18)	0.96–10.04
Progesterone (ng/mL)	4.05 ± 6.47	0.30 (0.10–4.30)	0.10–27.90
Prolactin (ng/mL)	18.92 ± 11.48	16.79 (11.43–22.58)	2.29–72.07

**Table 2:** Correlations for Untransformed (Raw) Variables with AMH.

Variable	Pearson’s r (95% CI)	*p*-Value	Spearman’s ρ (95% CI)	*p*-Value
Age	−0.376 (−0.522, −0.209)	<0.001	−0.317 (−0.480, −0.133)	<0.001
FSH	−0.445 (−0.580, −0.287)	<0.001	−0.503 (−0.634, −0.344)	<0.001
LH	0.139 (−0.044, 0.313)	0.134	0.057 (−0.137, 0.246)	0.539
Estradiol	−0.115 (−0.290, 0.068)	0.218	0.007 (−0.185, 0.199)	0.943
Progesterone	0.058 (−0.125, 0.237)	0.533	0.048 (−0.146, 0.238)	0.610
Prolactin	−0.053 (−0.232, 0.130)	0.572	0.049 (−0.145, 0.239)	0.601

**Table 3:** Correlations for Box–Cox-Transformed Variables with AMH_bc (λ = 0.309).

Variable	Pearson’s r (95% CI)	*p*-Value	Spearman’s rho (95% CI)	*p*-Value
Age	−0.33 (−0.483, −0.158)	<0.001	−0.317 (−0.480, −0.133)	<0.001
FSH_bc (λ = −0.358)	−0.454 (−0.587, −0.297)	<0.001	−0.503 (−0.634, −0.344)	<0.001
LH_bc (λ = 0.207)	0.061 (−0.122, 0.240)	0.516	0.057 (−0.136, 0.247)	0.539
Estradiol_bc (λ = −0.484)	0.048 (−0.135, 0.228)	0.607	0.007 (−0.186, 0.199)	0.943
Progesterone_bc (λ = −0.206)	0.049 (−0.133, 0.229)	0.598	0.048 (−0.146, 0.238)	0.610
Prolactin_bc (λ = 0.064)	0.016 (−0.166, 0.197)	0.862	0.049 (−0.145, 0.239)	0.601

**Table 4:** Multivariable Tobit Regression Model for Box–Cox-Transformed AMH (λ = 0.309).

Predictor Variable	β	95% CI	Std. Error	z-Value	*p*-Value	β*
(Intercept)	11.761	[5.703, 17.818]	3.091	3.805	<0.001	−0.040
Age (Years)	−0.068	[−0.117, −0.018]	0.025	−2.681	0.007	−0.234
FSH_bc (λ = −0.358)	−3.582	[−4.846, −2.317]	0.645	−5.553	<0.001	−0.559
LH_bc (λ = 0.207)	0.377	[−0.067, 0.821]	0.227	1.665	0.096	0.146
Estradiol_bc (λ = −0.484)	−2.773	[−5.608, 0.062]	1.447	−1.917	0.055	−0.192
Progesterone_bc (λ = −0.206)	0.011	[−0.133, 0.155]	0.074	0.151	0.880	0.013
Prolactin_bc (λ = 0.064)	−0.089	[−0.509, 0.330]	0.214	−0.417	0.677	−0.036

β: Unstandardized Estimate; β*: Standardised Estimate; CI: Confidence Interval. Model Diagnostics: Analytical sample *n* = 117; left−censored observations at analytical floor (0.02 ng/mL, BC_AMH_ = −2.270) = 15 (12.82%); uncensored = 102; scale = 1.446; log−likelihood = −220.7 on 8 Df; Wald statistic = 47.52 on 6 Df (*p* < 0.001). Dependent variable: AMH_bc (λ = 0.309).

**Table 5:** Sensitivity Analysis.

Model Specification	Included *n*	β Age (95% CI)	β FSH_bc (95% CI)	Key Methodological Findings/Interpretation
Primary Model (Table 4)	117	−0.068 (−0.117, −0.018)	−3.582 (−4.846, −2.317)	Baseline harmonised model (λ = 0.309; left-censored *n* = 15)
Including AMH > 10 ng/mL	124	−0.087 (−0.138, −0.037)	−3.874 (−5.196, −2.552)	Includes extreme cases; LH_bc becomes significant (*p* = 0.005)
Log-transformed AMH	117	−0.064 (−0.127, −0.001)	−3.767 (−5.364, −2.170)	Standard log transformation over-compresses scale (p_Age_ = 0.048)
Non-linear Age (RCS)	117	−0.125 (−0.238, −0.013)	−3.571 (−4.832, −2.311)	3-knot RCS model; LRT vs. linear *p* = 0.2663 (linear preferred)

Notes: CI: Confidence Interval; RCS: Restricted Cubic Spline; LRT: Likelihood Ratio Test.

## Data Availability

The deidentified individual-level dataset analysed in this study is available from the corresponding author upon reasonable request, subject to approval by the Istanbul Atlas University Ethics Committee. Aggregate and excluded-patient data are provided in Supplementary Tables S1–S4.

## Supplementary Materials

The following supporting information can be downloaded at https://www.mdpi.com/article/10.3390/metabo16090610/s1, Supplementary Table S1. Correlations among model predictors and variance inflation factors; Supplementary Table S2. Clinical and Endocrine Profile of Excluded High AMH Patients (AMH > 10 ng/mL); Supplementary Table S3. Estimated Box–Cox transformation parameters (λ) with 95% confidence intervals for each hormone; Supplementary Table S4. Standardised coefficients and average marginal effects for the multivariable Tobit model; Supplementary Figure S1. Residual diagnostic plots for the multivariable Tobit model.
