# Supplementary material for: Age and Follicle-Stimulating Hormone as Variables Independently Associated with Serum Anti-Müllerian Hormone in Women Attending a Tertiary Gynaecology Clinic: A Retrospective Cross-Sectional Study Using Censoring-Aware Modelling
Source: Metabolites. 2026 Aug 26;16(9):610. doi: 10.3390/metabo16090610 (PMC13609734; doi:10.3390/metabo16090610)
Supplement: Supplementary file 1 [file metabolites-16-00610-s001.zip › metabolites-4500108-supplementary.pdf]

SUPPLEMENTARY MATERIAL

Supplementary Table S1. Correlations among model predictors and variance inflation factors

Predictor	Age	FSH_bc	LH_bc	Estradiol_bc	Progesterone_bc	Prolactin_bc	VIF
Age	1.000	0.146	-0.069	0.042	-0.135	-0.042	1.075
FSH_bc	-	1.000	0.205	-0.442	-0.100	-0.038	1.349
LH_bc	-	-	1.000	-0.096	-0.032	0.136	1.078
Estradiol_bc	-	-	-	1.000	0.243	-0.038	1.339
Progesterone_bc	-	-	-	-	1.000	-0.126	1.106
Prolactin_bc	-	-	-	-	-	1.000	1.044

Supplementary Table S2. Clinical and Endocrine Profile of Excluded High AMH Patients (AMH > 10 ng/mL)

Patient ID	Age (Years)	AMH (ng/mL)	FSH (mIU/mL)	LH (mIU/mL)	Estradiol (pg/mL)
1386	31.4	16.4	4.77	4.66	43
1114	30.9	16	4.7	5.73	43
1590	23.7	15.1	4.95	20.3	55
204	26.1	14.1	4.81	7.31	42
1179	26.8	10.5	4.63	3.8	210
1536	23.5	10.2	5.39	9.94	44
1262	26.2	10	6.62	8.5	72

Supplementary Table S3. Estimated Box–Cox transformation parameters (λ) with 95% confidence intervals for each hormone.

Variable	Raw Mean ± SD	Skewness (Raw)	Optimal λ Estimate	95% Confidence Interval for λ	Transformed Variable Name
AMH	2.22 ± 2.24	1.136	0.309	[0.208, 0.416]	AMH_bc
FSH	7.53 ± 4.61	1.920	-0.358	[-0.642, -0.075]	FSH_bc
LH	4.45 ± 2.03	0.785	0.207	[-0.125, 0.552]	LH_bc
Estradiol	109.21 ± 226.20	4.631	-0.484	[-0.657, -0.321]	Estradiol_bc
Progesterone	4.05 ± 6.47	1.739	-0.206	[-0.332, -0.083]	Progesterone_bc
Prolactin	18.92 ± 11.48	2.275	0.064	[-0.157, 0.290]	Prolactin_bc

Notes: Chronological age remained untransformed (λ = 1.0). Optimal λ parameters were estimated via Maximum Likelihood Estimation across (MLE) evaluated over a fine grid (− 2.000 to + 2.000 in 0.001 increments) to maximize profile log-likelihood. The 95% Confidence Intervals were derived from profile log-likelihood bounds ($\chi^2_{1,0.95}/2 \approx 1.921$).

Supplementary Table S4. Standardised coefficients and average marginal effects for the multivariable Tobit model.

Predictor	β	β*	AME	Std. Error (AME)	z-value	p-value
(Intercept)	11.761	-0.040	10.854	2.852	3.805	< 0.001
Age (Years)	-0.068	-0.234	-0.063	0.023	-2.681	0.007
FSH_bc (λ = -0.358)	-3.582	-0.559	-3.306	0.595	-5.553	< 0.001

LH_bc (λ = 0.207)	0.377	0.146	0.348	0.209	1.665	0.096
Estradiol_bc (λ = -0.484)	-2.773	-0.192	-2.559	1.335	-1.917	0.055
Progesterone_bc (λ = -0.206)	0.011	0.013	0.010	0.068	0.151	0.880
Prolactin_bc (λ = 0.064)	-0.089	-0.036	-0.082	0.198	-0.417	0.677

Notes: β: Unstandardized Estimate, β^*: Standardized Estimate, AME: Average Marginal Effect. *Notes: β: Unstandardized Estimate (latent scale);* β^*: Standardized Estimate; AME: Average Marginal Effect on the observed outcome E[Y| X]; Average censoring adjustment scaling factor Φ(·) = 0.9229.

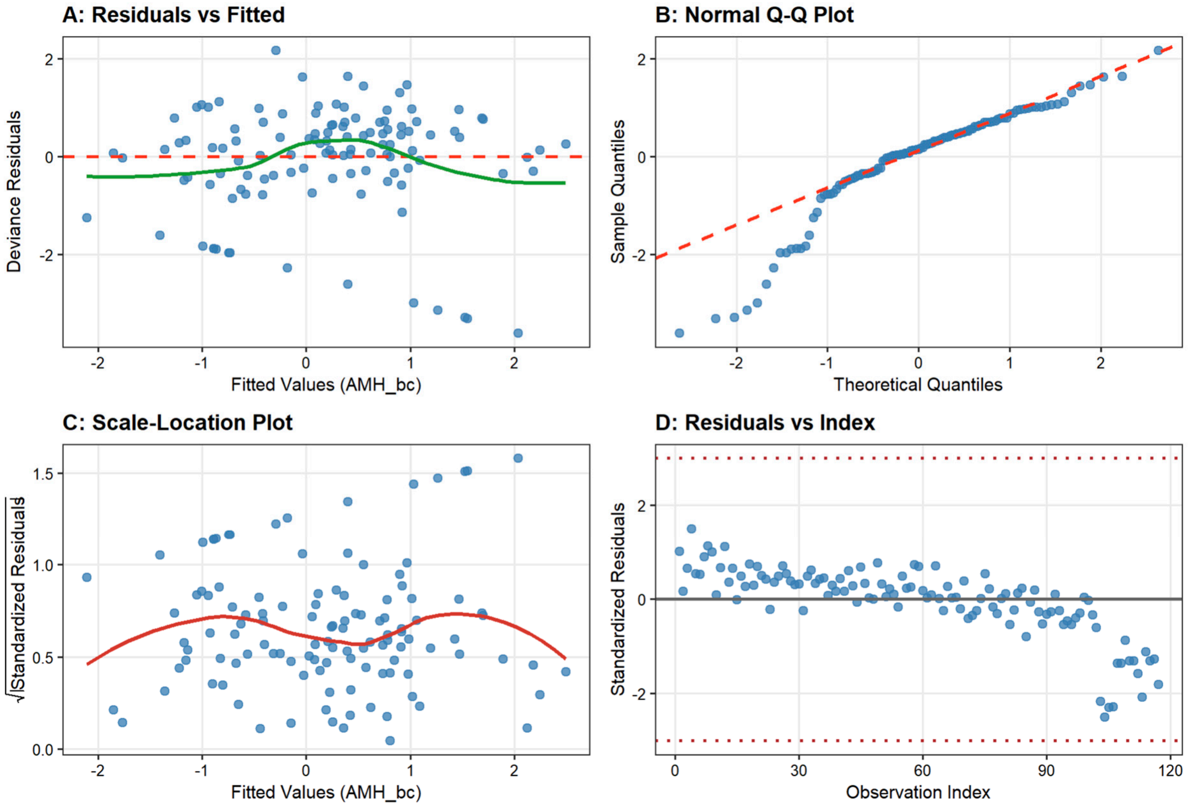

Supplementary Figure S1. Residual diagnostic plots for the multivariable Tobit model.
